# Effect of Pea Legumin-to-Vicilin Ratio on the Protein Emulsifying Properties: Explanation in Terms of Protein Molecular and Interfacial Properties

**DOI:** 10.1021/acs.jafc.3c01589

**Published:** 2023-07-11

**Authors:** Maud G. J. Meijers, Marcel B. J. Meinders, Jean-Paul Vincken, Peter A. Wierenga

**Affiliations:** † TiFN, Nieuwe Kanaal 9A, 6709 PA Wageningen, The Netherlands; ‡ Laboratory of Food Chemistry, Wageningen University and Research, Bornse Weilanden 9, 6708 WG Wageningen, The Netherlands; § Food and Biobased Research, Wageningen University and Research, Bornse Weilanden 9, 6708 WG Wageningen, The Netherlands

**Keywords:** plant protein, legumes, pea protein, interfacial properties, emulsion properties, emulsion droplet size

## Abstract

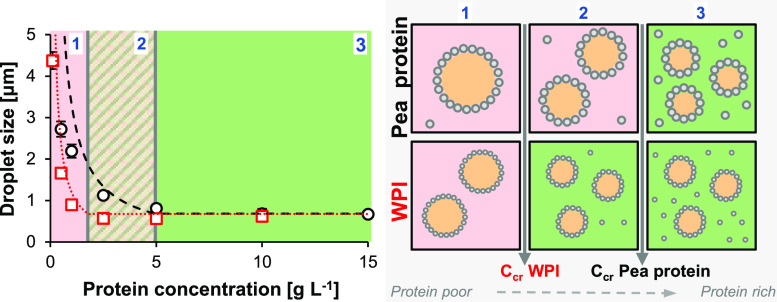

In isolates from different pea cultivars, the legumin-to-vicilin (L:V) ratio is known to vary from 66:33 to 10:90 (w/w). In this study, the effect of variations in the L:V ratio on the pea protein emulsifying properties (emulsion droplet size (*d*_3,2_) vs protein concentration (*C*_p_)) at pH 7.0 was investigated using a purified pea legumin (PLF_sol_) and pea vicilin fraction (PVF_sol_). Despite a different Γ_max,theo_, the interfacial properties at the oil–water interface and the emulsifying properties were similar for PLF_sol_ and PVF_sol_. Hence, the L:V ratio did not affect the pea protein emulsifying properties. Further, PLF_sol_ and PVF_sol_ were less efficient than whey protein isolate (WPI_sol_) in stabilizing the emulsion droplets against coalescence. This was explained by their larger radius and thus slower diffusion. For this reason, the difference in diffusion rate was added as a parameter to the surface coverage model. With this addition, the surface coverage model described the *d*_3,2_ versus *C*_p_ of the pea protein samples well.

## 1 Introduction

Widely varying results for emulsifying properties of pea protein samples have been reported. The emulsifying activity indices (EAI) determined for pea protein isolates (PPI), obtained with similar isolation methods and measured under similar conditions, range from 25 to 117 m^2^ g^–1^.^1−3^ Can these differences be explained by the pea protein composition? The ratio between the main globular proteins in pea, legumin, and vicilin is known to vary from 66:33 to 10:90 (w/w) depending on the cultivar.^3−8^ This study aims to determine the effect of the legumin-to-vicilin (L:V) ratio on the emulsifying properties by studying the emulsion droplet size (*d*_3,2_) as a function of protein concentration (*C*_p_) at pH 7.0. The experimental results were used to test if the recently developed model to predict the *d*_3,2_(9) could be used to describe the emulsifying properties of the pea protein mixtures.

To characterize the emulsifying properties of proteins, most studies report the emulsifying activity index (EAI) and/or the emulsifying capacity (EC). The emulsifying capacity is measured by adding oil to a protein solution while homogenizing until the point that phase inversion occurs.^10^ However, this point is the same, 57% (v/v), for all monomodal emulsions as already explained by Halling et al.^11^ The other parameter, the EAI, is defined as the amount of surface area (m^2^) formed per gram of total protein.^12,13^ The EAI is protein concentration-dependent; nonetheless, it is usually only measured at one protein concentration. Therefore, neither the EC nor the EAI provides information on the efficiency of a protein to stabilize an emulsion and/or allows comparison between different samples.

Contrary to the methods described above, Tcholakova et al.^14^ quantitatively described the effect of the ratio between protein concentration and oil fraction (*C*_p_/Φ_oil_) on the emulsion droplet size. They assumed that after the homogenizer valve, the newly formed emulsion droplets coalesced in case the amount of proteins that adsorbed within the timescale of homogenization was not sufficient to stabilize the droplets. The authors distinguished between a protein-rich and protein-poor regime. In the protein-rich regime, the emulsion droplet size does not decrease further with increasing protein concentration. The regimes are separated by the critical protein concentration (*C*_cr_). To compare the emulsifying properties of proteins at a given Φ_oil_, this *C*_cr_ should be determined. From data of the *d*_3,2_ versus *C*_p_ of β-lactoglobulin, at pH 7.0, 10 mM sodium phosphate, and Φ_oil_ = 0.1,^15^ a *C*_cr_ between 2.5 and 5 g L^–1^ protein was derived. Under similar conditions, the *C*_cr_ of commercial PPI, was two times higher: between 5 and 10 g L^–1^ protein.^16^ Hence, this commercial PPI was less efficient in stabilizing the newly formed emulsion droplets against coalescence than β-lactoglobulin. The *C*_cr_ of PPI may vary with the L:V ratio, but information on the effect of the L:V ratio on the *C*_cr_ is missing. However, values have been reported for the *d*_3,2_ of *pure* pea legumin or vicilin isolate emulsions at a single protein concentration. One study showed that pea legumin emulsions have a smaller mean droplet diameter than pea vicilin emulsions: 5.4 and 23.6 μm, respectively, at *C*_p_ 1.0 g L^–1^, pH 7.0, *I* = 0.08 M, and Φ_oil_ = 0.14.^17^ In contrast, another study showed no difference in the emulsion droplet size stabilized by pea legumin or vicilin: 3.6 and 3.2 μm, respectively, at *C*_p_ 10 g L^–1^, pH 7.0, deionized water, and Φ_oil_ = 0.1.^18^ There is a generally held idea that pea vicilin has better interfacial and emulsifying properties than pea legumin due to its more flexible structure.^19,20^ However, the published droplet sizes of the pea legumin and vicilin stabilized emulsions appear seemingly contradictory and therefore do not support this idea. It should be recognized that the studies have a different *C*_p_/Φ_oil_ ratio.

The *C*_cr_, as described above, describes the ability of a protein to stabilize an emulsion droplet against coalescence within the timescale of homogenization. Another property often used in the description of emulsions is the stability against flocculation, which also depends on the protein concentration. The protein concentration at which the emulsion droplet is stable against coalescence (*C*_cr_) is not necessarily the same as the concentration where it is stable against flocculation. The degree of flocculation is often described by the flocculation index (FI), which is calculated by dividing the flocculate size by the individual emulsion droplet size minus 1. Flocculation was reported for emulsion droplets stabilized by commercial PPI at *C*_p_ < 10 g L^–1^, 10 mM phosphate buffered solution, Φ_oil_ = 0.1.^16^ In addition, flocculation was reported for emulsion droplets stabilized by non-commercial PPI, pea legumin isolate and pea vicilin isolate: FI = 4.36, 4.30, and 5.56, respectively, at *C*_p_ 10 g L^–1^, pH 7.0, and Φ_oil_ = 0.1.^18^ The flocculation of a commercial PPI, with protein low solubility, was determined at protein concentrations from 1 to 30 g L^–1^. Interestingly, the FI was found to be protein concentration-dependent. The highest FI was found at the lowest protein concentration: FI = 3.24 ± 0.83, *C*_p_ 1 g L^–1^, 10 mM phosphate buffered solution, and Φ_oil_ = 0.1.^16^ This effect of protein concentration on the emulsion flocculation behavior was explained, by other authors, as a stabilizing effect of excess protein in the bulk, by the addition of a repulsive force or the possibility of a higher maximum adsorbed amount of protein (Γ_max_).^15^

In this study, the effect of the pea L:V ratio on the protein emulsion properties was studied by measuring the emulsion particle and droplet and flocculate size (*d*_3,2_) versus the protein concentration (*C*_p_). Pea protein concentrate (PPC), pea legumin fraction (PLF), pea vicilin fraction (PVF), a blend of PLF and PVF, and whey protein isolate (WPI) were studied. The recently developed surface coverage model^9^ was extended and used to predict the *C*_cr_ of the protein samples.

## 2 Materials and Methods

### 2.1 Materials

Yellow peas (*Pisum sativum* Leguminosae) were purchased from Alimex Europe B.V. (Sint-Kruis, Belgium). BiPro, a commercial whey protein isolate (WPI), was obtained from Davisco Foods International Inc. Rapeseed oil was provided by Danone Nutricia (Utrecht, The Netherlands). SDS-PAGE Precision Plus Protein marker, Mini-PROTEAN TGX precast gels, Zymogram sample buffer, and Tris/glycine/SDS running buffer were purchased from Bio-Rad Laboratories (Hercules, CA, USA). Coomassie blue stain was purchased from Expedeon (San Diego, CA, USA). All other chemicals were of analytical grade and purchased from either Merck (Darmstadt, Germany), Sigma-Aldrich (St. Louis, MO, USA), or Acros Organics (Geel, Belgium). All water was obtained from a Milli-Q system (Millipore, Billerica, MA, USA). The diluted McIlvaine buffer was prepared by mixing 20 mM disodium phosphate and 10 mM citric acid, both dissolved in Milli-Q (MQ) water, until pH 7.0 was reached.

### 2.2 Methods

#### 2.2.1 Protein Isolation from Yellow Pea

PPC was prepared by alkaline extraction followed by iso-electric precipitation, as described by Vreeke et al.^8^ Whole frozen peas (Alimex) were broken with a pin mill (LV 15M Condux-Werk, Wolfgang bei Hanau, Germany) and subsequently milled (ZPS50 impact mill, Hosokawa-Alpine, Augsburg, Germany). The pea flour (10%, w/w) was suspended in Milli-Q water (MQ). The suspension was adjusted to pH 8.0, followed by centrifugation (17,000 × *g*, 4 °C, 20 min). The supernatant was collected and adjusted to pH 4.5, followed by centrifugation (17,000 *× g*, 4 °C, 20 min). The pellet was recovered and suspended in MQ at a final concentration of 10% (w/w, wet pellet) and adjusted to pH 8.0. The obtained solution was centrifuged (17,000 *× g*, 4 °C, 20 min), and the resulting supernatant was frozen (PPC^–20^), freeze-dried, and named PPC. Prior to all centrifugation steps, suspensions and solutions were kept at 4 °C and the set pH while being stirred for at least 2 h.

#### 2.2.2 Legumin and Vicilin Fractionation from PPC

The PPC^–20^ was further fractionated to obtain PLF and PVF, as described by Vreeke et al.^8^ Defrosted PPC^–20^ was adjusted to pH 8.0 with NaOH and stirred for 1 h at 4 °C. The solution was subsequently diluted 1:1 with a McIlvaine buffer of pH 4.8, to a final concentration of 200 mM disodium phosphate and 100 mM citric acid containing 200 mM NaCl. The sample was stirred at 4 °C for at least 2 h, followed by centrifugation (17,000 *× g*, 4 °C, 20 min). The obtained supernatant containing the pea vicilin was filtered using an ultrafiltration system with a 5 kDa membrane (Hydrosart Ultrafilter, Sartorius AG, Frankfurt, Germany). The liquid removed during ultrafiltration was replenished by MQ. The retentate, rich in pea vicilin, was frozen, freeze-dried, and named PVF. The legumin-rich pellet was resuspended in 20.0 mM Tris-HCl buffer, pH 8.0, (buffer A) at a final concentration of approximately 10 g L^–1^. The solution was stirred for at least 2 h, prior to centrifugation (17,000 *× g*, 4 °C, 20 min). The obtained supernatant was filtered over a glass fiber pre-filter (13400-142-K, Sartorius) with a Whatman filter paper (black ribbon, 589/1, GE Healthcare, Uppsala, Sweden). The filtrate was applied onto a Source 15Q column (Fineline, Pfizer Manufacturing, Freiburg, Germany) coupled to an ÄKTA explorer system (GE Healthcare). Elution was similar to the method as described by O’Kane et al.^21^ and fractions were collected. The fractions rich in legumin were pooled and filtered using an ultrafiltration system with a 5 kDa membrane (Hydrosart Ultrafilter, Sartorius AG). The liquid removed during ultrafiltration was replenished by MQ. The retentate, rich in pea legumin, was frozen, freeze-dried, and named PLF.

#### 2.2.3 Preparation of PPC_sol_, PLF_sol_, PVF_sol_, and WPI_sol_ for Analysis

PPC, PLF, PVF, and WPI were dissolved at 25.0 g protein L^–1^ in the diluted McIlvaine buffer (20 mM disodium phosphate and 10 mM citric acid), pH 7.0. The samples were stirred for at least two hours at room temperature (RT) after which they were centrifuged (4696 × *g*, 10 min, 20 °C). The supernatants were collected and called PPC_sol_, PLF_sol_, PVF_sol_, and WPI_sol_. In further experiments, the soluble fractions were used unless stated otherwise. The protein and carbohydrate composition of the soluble samples were similar to those of the respective total samples. The differences in the protein solubility were corrected by normalizing the soluble protein concentration. Legumin-vicilin blends were prepared by mixing PLF_sol_ and PVF_sol_ in the following ratios (V/V) 70:30 (LV_7030_), 50:50 (LV_5050_), 30:70 (PPC_sim_). PPC_sim_ contained legumin and vicilin in the same ratio as PPC. The two samples were compared to elucidate the effect of the non-protein fraction in PCC on the interfacial and emulsion properties.

#### 2.2.4 Preparation of the Stripped Rapeseed Oil

Rapeseed oil was stripped according to the protocol described by Berton et al.^22^ This protocol is an adapted version of the protocol originally developed by Maldonado-Valderrama et al.^23^ The oil (30 mL) and 15 mL of silica (Florisil, Sigma Aldrich) were mixed in 50 mL polypropylene centrifugal tubes, which were rotated overnight at 4 °C, without light exposure. Afterward, the tubes were centrifuged (2000 × *g*, 20 min, 20 °C), and the supernatant was collected and centrifuged again (2000 × *g*, 20 min, 20 °C). The supernatants were stored at −20 °C prior to use.

#### 2.2.5 Compositional Analysis

##### 2.2.5.1 Total Nitrogen Content and Protein Solubility

The total nitrogen content was determined in triplicate using the Dumas method (Flash EA 1112 N analyzer, Thermo Fisher Scientific, Waltham, MA, USA), according to the manufacturer’s protocol. A calibration curve of methionine (1.00–20.00 mg) was used for the nitrogen quantification. For WPI, a nitrogen conversion factor of 6.32 was used.^24^ For the pea protein samples, a nitrogen conversion factor of 5.4 was used, as calculated from the average nitrogen conversion factor of the following pea protein genotypes legumin A (P02857, UniProt Database), legumin J (P05692, UniProt Database), legumin A2 (P15838, UniProt Database), legumin K (P05693, UniProt Database), legumin B (P14594, UniProt Database), and vicilin (P13918, UniProt Database).^25^ The solubility of PPC, PLF, PVF, and WPI was determined at 25.0 g L^−1^ protein in the diluted McIlvaine, pH 7.0. The samples were prepared in duplicate, on which triplicate measurements were performed. The solubility was determined by dividing the protein content of the dried soluble sample by the protein content of the dried total sample multiplied by 100. The samples were dried at 60 °C overnight.

##### 2.2.5.2 Carbohydrate Composition

The neutral sugar composition of PPC, PLF, PVF, PPC_sol_, PLF_sol_, and PVF_sol_ was analyzed in duplicate after pre-hydrolysis with 72% (w/w) H_2_SO_4_ (1 h, 30 °C) followed by further hydrolysis with 1 M H_2_SO_4_ (3 h, 100 °C) using inositol as an internal standard. The monosaccharides released were derivatized and analyzed as their alditol acetates by gas chromatography.^26^ Arabinose, galactose, glucose, fucose, mannose, rhamnose, and xylose were used as standards. The uronic acid content was determined in duplicate by the automated colorimetric *m*-hydroxydiphenyl method^27^ on a Skalar auto-analyzer (Skalar Analytical B.V., Breda, The Netherlands). Samples were pre-hydrolyzed as described in the neutral carbohydrate composition method. Galacturonic acid (0–100 μg mL^–1^) was used for calibration.

##### 2.2.5.3 Moisture Content

The moisture contents of PPC, PLF, and PVF were determined gravimetrically by drying approximately 10 mg at 105 °C overnight.^28^ The measurements were performed in triplicate.

##### 2.2.5.4 Ash Content

The ash content of the samples was determined gravimetrically by a previously published method,^29^ with modifications. Approximately, 7 mg of the sample was dried at 105 °C overnight and subsequently incinerated at 525 °C overnight. The measurements were performed in triplicate. The ash content of PPC, PLF, and PVF was found to be different. Therefore, the contribution of the ash to the conductivity of the samples was estimated. A calibration curve of 5, 10, 20, 50, 100, and 500 mM NaCl solutions was prepared in duplicate. The conductivity was measured and plotted against the NaCl concentration. The equivalent NaCl concentration in the samples was calculated based on the ash content of the samples, assuming all ash was NaCl. Using the equivalent NaCl concentration and the calibration curve, the absolute contribution of the ash in the sample to the conductivity was calculated. This was expressed as a relative contribution to the total conductivity by dividing the conductivity of the ash by the conductivity of the ash plus the conductivity of the buffer. The conductivity of the buffer was determined experimentally.

#### 2.2.6 Protein Composition

##### 2.2.6.1 SDS-PAGE

The protein composition of PPC, PLF, PVF, PPC_sol_, PLF_sol_, LV_7030_, LV_5050_, PPC_sim_, and PVF_sol_ was determined using SDS-PAGE in the presence and absence of a reducing agent. All samples were diluted to 3 g L^–1^ protein in the diluted McIlvaine buffer, pH 7.0, and analyzed according to the manufacturer’s protocol. The samples were applied to gels (any kD, Mini-protean TGX precast protein gels, Bio-Rad Laboratories) and separated on a Miniprotean II system (Bio-Rad Laboratories). The proteins were stained with Coomassie blue stain (InstantBlue, Expedeon). The gels were scanned and analyzed using a densitometer (GS-900, Bio-rad laboratories) and Image Lab software (Bio-Rad laboratories). The relative protein composition was determined by averaging the annotated bands under reducing and non-reducing conditions. Under reducing conditions, the following bands were annotated: ∼93 kDa lipoxygenase,^30^ ∼70 kDa convicilin, ∼50 kDa vicilin, ∼38–40 kDa legumin acidic polypeptide, ∼33 and 30 kDa vicilin αβ and βγ fragments, ∼19–22 kDa legumin basic polypeptide, and ∼19, 16, and 13.5 kDa vicilin α, β, and γ fragments.^31^ Under non-reducing conditions, legumin was present as a monomer consisting of an acidic and basic polypeptide chain, therefore bands of ∼57–62 kDa were ascribed to legumin.^31^ The intensity of all unidentified bands was summed, and the total was referred to as “other proteins”.

##### 2.2.6.2 Size-Exclusion Chromatography (SEC)

The protein molecular weight distribution was determined using an ÄKTA PURE M25 (GE Healthcare). PPC_sol_, PLF_sol_, LV_7030_, LV_5050_, PPC_sim_, PVF_sol_, and WPI_sol_ were diluted to a protein concentration of 10.0 g L^–1^ in the diluted McIlvaine buffer, pH 7.0. The samples were centrifuged (16,100 × *g*, 10 min, 20 °C), and subsequently 50 μL of supernatant was injected onto a Superdex 200 Increase 10/300 GL (GE Healthcare). The samples were eluted using the diluted McIlvaine buffer with 150 mM NaCl at a flow rate of 0.5 mL min^–1^. The absorbance was measured at 280, 214, and 220 nm. Ferritin (474 kDa), aldolase (158 kDa), conalbumin (75 kDa), ovalbumin (44 kDa), carbonic anhydrase (29 kDa), and ribonuclease (13.7 kDa) were used as calibration standards.

#### 2.2.7 Determination of the ζ-Potential

PPC_sol_, PLF_sol_, LV_7030_, LV_5050_, PPC_sim_, PVF_sol_, and WPI_sol_ were diluted to a concentration of 10.0 g L^–1^ in the diluted McIlvaine buffer, pH 7.0. The ζ-potential of the samples was measured using the Zetasizer Nano ZSP (Malvern Instruments, Worcestershire, UK), as described previously.^15^ The measurements were performed at 40 V and at 25 °C, after equilibrating the system for 2 min. The ζ-potential measurements were performed on duplicate samples. For each sample, at least five sequential readings were carried-out. The ζ-potentials were calculated with Henry’s equation using the Smoluchowski approximation.^32^

#### 2.2.8 Quantification of Exposed Hydrophobicity

The protein’s exposed hydrophobicity was determined using 8-anilino-1-naphthalenesulfonic acid (ANSA) as a fluorescent probe, as previously described^33^ with adaptations. PPC_sol_, PLF_sol_, LV_7030_, LV_5050_, PPC_sim_, PVF_sol_, and WPI_sol_ were diluted to protein concentrations of 1.0, 2.0, 2.5, 5.0, and 10.0 g L^–1^ in the diluted McIlvaine buffer, pH 7.0. ANSA was dissolved at 0.8 mM in the same buffer and 20 μL ANSA solution was added to 200 μL protein solution. The samples were prepared in duplicate after which duplicate measurements were performed using a SpectraMax iD3 microplate reader (Molecular Devices, San Jose, CA, USA). For excitation, light with a wavelength of 385 nm was used. The emission spectrum was measured from 400 to 650 nm at 25 °C. The emission and excitation slits were set to 5 nm. The areas of the samples were corrected with the area of the buffer and plotted against the protein concentration. The relative exposed hydrophobicity was expressed as the slope of the linear regime obtained for the sample relative to that obtained for WPI_sol_.

#### 2.2.9 Adsorption Kinetics and Surface Elastic Modulus

The surface tension and surface elastic modulus were measured as a function of time at 20 °C in duplicate, using an automated drop tensiometer (Teclis Tracker, Teclis IT Concept, Longessaigne, France), according to the method described by Delahaije et al.^34^ PPC_sol_, PLF_sol_, LV_7030_, LV_5050_, PPC_sim_, PVF_sol_, and WPI_sol_ were diluted to a protein concentration of 0.05 g L^–1^ in the diluted McIlvaine buffer, pH 7.0. The stripped rapeseed oil was used for the oil-in-water measurements. For the air-in-water measurements, the air drop, rising from a curved needle (G18, Teclis) was kept constant at 20 mm^2^. For the oil-in-water measurements, the oil drop, rising from a curved needle (G20, Teclis) was kept constant at 30 mm^2^. The surface pressure at the air-buffer and oil-buffer interface, without protein, was measured for one hour to validate that the system was clean. The surface tension (γ [mN m^–1^]) can be expressed as surface pressure (Π [mN m^–1^]), which is the change in surface tension compared to that of a pure air–water or oil–water interface. The elastic modulus (*E*_d_) was measured by inducing sinusoidal changes in the interfacial area with an amplitude of 5% and a frequency of 0.1 Hz. Every 100 s, a sequence of five sinuses was performed. The change in surface tension and area averaged over sinus 2–5, per sequence of five sinuses, was used to determine the elastic modulus.

#### 2.2.10 Emulsion Preparation

To be able to determine the critical protein concentration (*C*_cr_), yet reduce the amount of samples to be measured, a high protein concentration sample was included for the pea protein samples and not for WPI_sol_. In addition, a low protein concentration sample was included for WPI_sol_ and not for the pea protein samples. Therefore, PPC_sol_, PLF_sol_, PPC_sim_, and PVF_sol_ were diluted to 0.5, 1.0, 2.5, 5.0, 10.0, and 15.0 g L^–1^ in the diluted McIlvaine buffer, pH 7.0. In addition, WPI_sol_ was diluted to 0.1, 0.5, 1.0, 2.5, 5.0, and 10.0 g L^–1^ in the diluted McIlvaine buffer, pH 7.0. Approximately 10% (v/v) of the stripped rapeseed oil was added to the protein solutions. The samples were pre-homogenized using an Ultra-Turrax (Type T-25B, IKA, Staufen, Germany) at 9500 rpm for 1 min. Afterward, the samples were passed 30 times through a homogenizer (Labhoscope HU-3.0, Perkin Elmer, Drachten, The Netherlands) set at 150 bar. During homogenization, the samples were cooled on ice water. The samples were stored for at least 15 h at 4 °C, without light exposure, prior to further analysis.

#### 2.2.11 Emulsion Particle Size Determination

The volume-surface average diameters (*d*_3,2_) of the emulsion droplets or flocculates were determined by static light scattering using a Mastersizer 3000 (Malvern Instruments Ltd., Malvern, UK) equipped with a Hydro SM sample dispersion unit. The refractive index used for water was 1.33. The refractive index of the stripped rapeseed oil was based on the reported value, 1.47, by Sridharan et al.^35^ This value was set at 1.473 by minimizing the residuals and weighted residuals in the fitted model. The samples were prepared and measured in duplicate. Each measurement was an average of at least five sequential readings at RT. The emulsions were measured “as is” and after diluting 1:1 in a 1% (w/v) SDS solution. The addition of the SDS allowed the measurement of the individual emulsion droplet size as the emulsion flocculates were dissociated.

#### 2.2.12 Flocculation Index

The FI of the samples was calculated using eq 1.
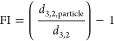
1Here, *d*_3,2,particle_ is the volume-surface average diameter of emulsion droplets/particles, and *d*_3,2_ of the individual emulsion droplets after diluting 1:1 in a 1% (w/v) SDS solution.

#### 2.2.13 Theoretical Prediction of Emulsion Droplet Size

The theoretical volume-surface average diameter (*d*_3,2,theo_) was calculated by extending the surface coverage model, as described by Delahaije et al.^9^ In short, the extended model takes into account the presence of multiple proteins with different properties (radius, charge, hydrophobicity). The relative diffusion coefficient was added to account for the difference in radii of the proteins present. The *d*_3,2,theo_ was calculated using eq 2.
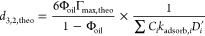
2Here, Φ_oil_ is the oil fraction (−), Γ_max,theo_ is the maximum adsorbed amount of protein on the interface (mg m^–2^), *C* is the protein concentration (g L^–1^), *k*_adsorb_ is the adsorption rate constant (−), and *D*′ is the relative diffusion coefficient (−). Previous research has shown that the *k*_adsorb_ of β-lactoglobulin, ovalbumin, and lysozyme at 10 mM sodium phosphate buffer, pH 7.0 can be approximated by the relative exposed hydrophobicity (*Q*_h_).^34^ This *Q*_h_ was calculated by dividing the exposed hydrophobicity of the sample by that of β-lactoglobulin. In this study, the *Q*_h_ of the samples was calculated by dividing the exposed hydrophobicity of the sample by that of WPI_sol_. When the *Q*_h_ of the sample was equal to or higher than the *Q*_h_ of WPI_sol_, it was assumed that there was no barrier for the adsorption of the protein to the interface. Hence, a value of 1 for *k*_adsorb_ was used. The relative diffusion coefficient was calculated by dividing the diffusion coefficient of legumin, aggregated legumin or vicilin by that of β-lactoglobulin. The diffusion coefficient was calculated using eq 3.
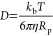
3Here, *k*_b_ is the Boltzmann constant (J K^–1^), *T* is the temperature (K), η is the viscosity of the medium (Pa s), and *R*_p_ is the radius of the protein (m).

Γ_max,theo_ was calculated taking into account the presence of different proteins, at different concentrations (eq 4).
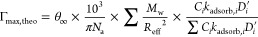
4Here, θ_∞_ is the saturation coverage, which has a value of 0.547 for random sequential adsorption of non-diffusing hard spherical particles.^36^*N*_a_ is Avogadro’s constant (6.022 × 10^23^ mol^–1^), *M*_w_ is the molecular weight of the protein (g mol^–1^), and *R*_eff_ is the effective radius of the protein (m) which was calculated from radius of the protein (*R*_p_) and the contribution to the radius by electrostatic forces. The *R*_p_ of WPI_sol_ and legumin aggregates was calculated theoretically assuming spherical particles with a specific *M*_w_.^9^ The *R*_p_ of the legumin hexamers and vicilin trimers was estimated from the crystal structure and 4.75 × 10^–9^ m was used for both.^37^ The effective radius (*R*_eff_) was calculated, as described by Delahaije et al.^9^ The constant in the calculation of the *R*_eff_ was assumed to be similar as that of β-lactoglobulin: 1.77 × 10^–9^, at pH 7.0.^34^

The *d*_3,2,theo_ vs *C*_p_ was calculated for PLF_sol,_ PLF_sol,aggr_, PPC_sol,_ PVF_sol_, and WPI_sol_. For WPI_sol_, the radius of β-lactoglobulin was used for the calculations. For the pea protein samples, it was assumed all protein was either legumin and/or vicilin, and the legumin-to-vicilin (L:V) ratio as determined by SDS-PAGE was used to estimate the composition. For PLF_sol,aggr_, the presence of 24% aggregated legumin (600 kDa) and 76% hexameric legumin, as measured by size-exclusion chromatography, was taken into account.

#### 2.2.14 Light Microscopy

The emulsions were analyzed by light microscopy using an Axioscope A01 (Carl Zeiss, Sliedrecht, The Netherlands) at 40× magnification. The samples were diluted 10× in the diluted McIlvaine buffer or a 1% (w/v) SDS solution in MQ to confirm the dissociation of emulsion flocculates. The samples were analyzed at RT.

## 3 Results and Discussion

### 3.1 Composition and Properties of the Protein Isolates

#### 3.1.1 Gross and Protein Composition

The protein contents of the pea protein concentrate (PPC, 75.0 ± 0.5%) and the pea vicilin fraction (PVF, 75.8 ± 1.5%) were similar to each other and that of the PLF was higher (PLF, 90.5 ± 1.0%) (Table 1). Surprisingly, the ∼15% higher protein content of PLF compared to PCC and PVF was not compensated by a similar decrease in the carbohydrate content: PLF 0.5 ± <0.1% compared to PPC 3.1 ± 0.2% and PVF 1.6 ± 0.2%, (w/w). Further, the difference in protein content was not likely explained by the lipid content: 2.2% (w/w dry matter) in the pea flour.^38^ The difference in protein contents could only in part be explained by the difference in ash content: PPC 6.6 ± 0.2%, PVF 11.4 ± 0.1%, PLF 2.0 ± <0.1% (w/w). The discrepancy between the increased protein contents and the decreased contents of other compounds (carbohydrates, lipids, ash) can be explained by the differences in total annotated dry matter. In total, 85, 89, and 93% of PPC, PVF, and PFL were annotated (w/w dry matter). Although some studies were able to annotate approximately 100% of the dry matter in pea,^39^ incomplete mass balances of plant extracts and concentrates are not uncommon.^28,38^ Often the amount of carbohydrates is calculated as 100% minus the sum of the other constituents quantified.^40−43^ Due to this calculation method incomplete mass balances are not visible.

**Table 1:** Protein Solubility (%) of the Pea Protein Samples at pH 7.0, in the Diluted McIlvaine Buffer (20 mM Disodium Phosphate and 10 mM Citric Acid) and Gross Chemical Composition of the Total Samples (%, w/w) on Dry Matter, Both ± Standard Deviation

component	PPC	PLF	PVF
protein solubility	94.1 ± 4.8	97.8 ± 0.4	98.9 ± 0.1
proteins	75.0 ± 0.5	90.5 ± 1.0	75.8 ± 1.5
carbohydrates	3.1 ± 0.2	0.5 ± <0.1	1.6 ± 0.2
neutral	2.6 ± 0.2	0.2 ± 0.1	0.8 ± 0.2
charged	0.5 ± <0.1	0.3 ± <0.1	0.7 ± <0.1
ash	6.6 ± 0.2	2.0 ± <0.1	11.4 ± 0.1
lipids	ND^a^	ND^a^	ND^a^
total annotated	85	93	89

a The oil content of the starting material (pea flour) has been previously determined and was only 2.2% (w/w).^38^

As described above, there were differences in the ash contents of the different samples. At low protein concentrations, such as for the interfacial measurements (PLF, PPC, PVF, 0.05 g L^–1^ protein), the ash coming from the sample contributed only 0.1–0.4% to the total conductivity in the solution. The amount of ash in the samples was therefore too small to affect the interfacial properties. At higher protein concentrations, mainly for the emulsion measurements in the protein-rich regime, this contribution was larger, (respectively 17, 45, and 57% for PLF, PPC, and PVF, 15.0 g L^–1^ protein). To exclude a possible effect of the ash content on the emulsion properties, an experiment was performed where NaCl was added to PLF to correct for the ash content. The results showed no differences in the emulsion properties of PLF with or without the addition of NaCl (data not shown).

**Figure 1:**
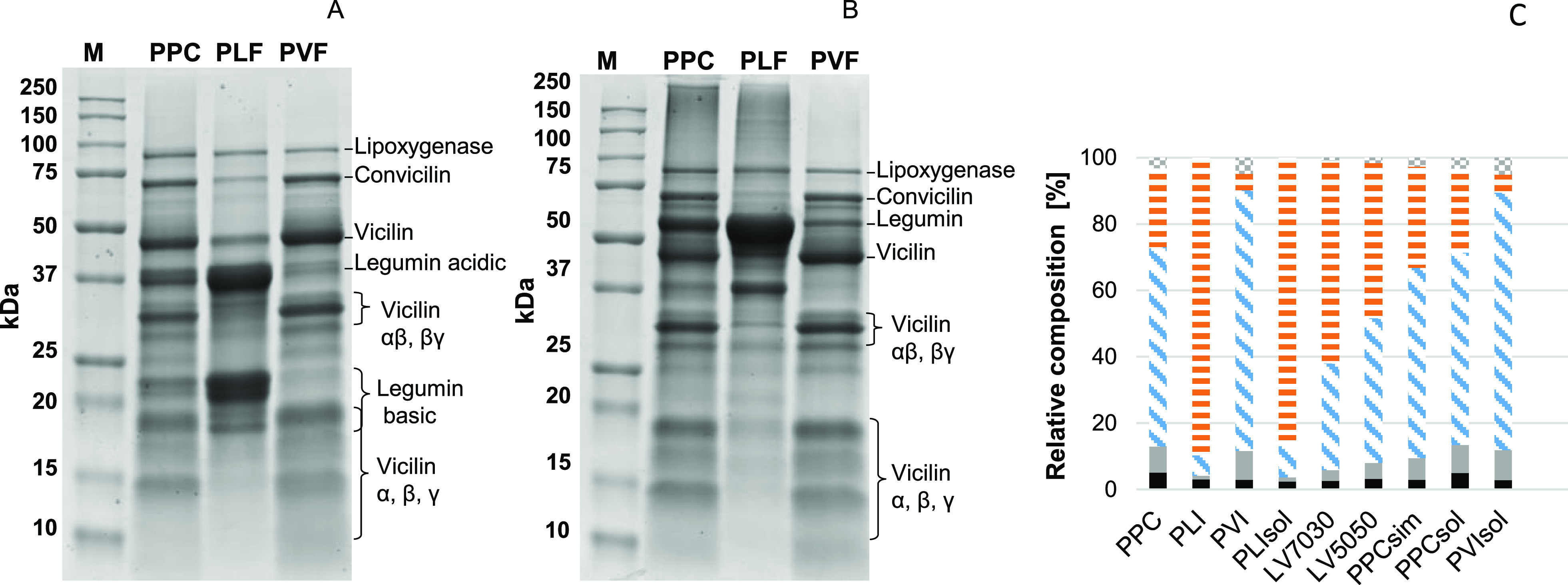
SDS-PAGE gels of PPI, PLF and PVF stained with Coomassie, under reducing conditions (A) and non-reducing conditions (B). M: molecular weight marker. Composition (%) of samples based on densitometry of SDS-PAGE gels showing legumin (horizontal orange lines), vicilin (diagonal blue lines), convicilin (gray boxes), lipoxygenase (black boxes) and other proteins (gray-white checkered boxes) (C).

The protein composition of PPC was further studied using SDS-PAGE. Based on densitometry, the protein part of PPC consisted of 24% legumin and 60% vicilin (w/w), yielding a legumin-to-vicilin (L:V) ratio of 28:72 (w/w, Figure 1). The protein in PPC was 94.1 ± 4.8% soluble (Table 1), and this soluble part (PPC_sol_) was used for further experiments. In PPC_sol_, the L:V ratio was quite similar (31:69, w/w) to the ratio in PPC. In PLF and PVF, legumin accounted for 90%, and vicilin for 79% of all proteins (w/w), respectively. In the soluble fractions, PLF_sol_ and PVF_sol_ (protein solubility: 97.8 ± 0.4 and 98.8 ± 0.1% of total protein, Table 1), this was quite similar, 87 and 78% (w/w), respectively. PLF_sol_ and PVF_sol_ were mixed in a 30:70 ratio (v/v, PPC_sim_) to obtain a similar L:V ratio as present in PPC_sol_. The L:V ratio of PPC_sim_ was 35:65 (w/w), which indeed was similar as in PPC_sol_ (31:69, w/w).

#### 3.1.2 Molecular Properties

**Figure 2:**
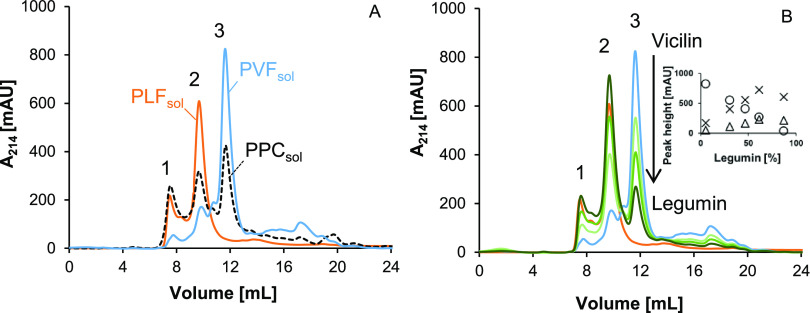
Size-exclusion chromatography elution patterns of PLF_sol_ (solid orange lines), PVF_sol_ (solid blue lines), PPC_sol_ (dashed black line) (A) and PLF_sol_, PVF_sol_, and blends (PPC_sim_ (solid light green line), LV_5050_ (solid medium green line), LV_7030_ (solid dark green line)) (B). Elution peaks consistent with a molecular weight of aggregated legumin indicated with number 1, consistent with hexameric legumin indicated with number 2, and consistent with trimeric vicilin indicated with number 3. Arrow in panel B indicates samples with decreasing vicilin and increasing legumin content. The inset shows the peak heights of peak 1 (black traingles), 2 (black crosses) and 3 (black circles) as function of the legumin fraction based on densitometry (%, w/w).

In PLF_sol_, 74% hexameric legumin (∼440 kDa) and 26% soluble, aggregated legumin were present (>600 kDa) based on size-exclusion chromatography (Figure 2A). The presence of legumin in its hexameric form, is in line with reported data in the literature.^44^ The presence of the legumin aggregates was a result of the system conditions rather than an effect of the isolation, as the aggregates disappeared at higher ionic strength (results not shown). No literature information is available on this effect. PVF_sol_ mainly consisted of trimeric vicilin (∼180 kDa, Figure 2A). Pea vicilin is known to occur as trimers in the pea seed.^45^ Due to the multimeric nature of pea legumin and vicilin, the radii of the proteins could not be estimated assuming the molecules were spherical. Therefore, the radii (*R*_p_) of pea legumin and vicilin were estimated from the crystal structure of the proteins: *R*_p_ = 4.750 × 10^–9^ m for both proteins. The *R*_p_ of WPI_sol_ was calculated from the theoretical molecular weight of β-lactoglobulin: *R*_p_ = 2.196 × 10^–9^ m. The *R*_p_ of WPI_sol_ was more than 2 times smaller than the radii of the pea legumin and vicilin, respectively. No differences were found between the ζ-potentials of the different samples. All samples had a high relative hydrophobicity of 1.0 or higher, resulting in an adsorption rate constant (*k*_adsorb_) of approximately 1 (Table 2). Therefore, the main difference in molecular properties amongst the proteins was the radii of the proteins. The chromatograms of the L:V mixtures, blended from PLF_sol_ and PVF_sol_, were the weighted averages of the chromatograms of the individual samples (Figure 2B). This confirmed that PLF_sol_ and PVF_sol_ did not form aggregates together under these conditions. The molecular properties of the proteins as described above: radius, ζ-potentials, and *k*_adsorb_ were used to estimate the volume–surface average diameter (*d*_3,2_) of the emulsion droplets. For the prediction of the *d*_3,2_, additive contributions of each protein in the mixture were assumed, since the proteins did not form aggregates.

**Table 2:** Protein Molecular Properties and Parameters for the Calculation of *d*_3,2,theo_

protein	*M*_w_ (Da)	|ζ-potential| (mV)^c^	*R*_eff_ (m)	relative hydrophobicity (−)^d^	*k*_adsorb_ (−)	diffusion coefficient (m^2^ s^–1^)	relative diffusion coefficient (−)	Γ_max,theo_ (mg m^–2^)
PLF_sol_	436,000^a^	15.0 ± 2.8	5.814 × 10^–9^	1.31 ± 0.08	1.00	4.15 × 10^–11^	0.424	3.52
PLF_sol,aggr_	N/A^e^	15.0 ± 2.8	N/A^e^	1.31 ± 0.08	1.00	N/A^e^	N/A^e^	3.58
LV_7030_	N/A^e^	14.8 ± 2.9	N/A^e^	1.34 ± 0.16	1.00	N/A^e^	N/A^e^	3.00
LV_5050_	N/A^e^	14.1 ± 3.3	N/A^e^	1.22 ± 0.20	1.00	N/A^e^	N/A^e^	2.69
PPC_sim_	N/A^e^	13.7 ± 2.1	N/A^e^	1.12 ± 0.16	1.00	N/A^e^	N/A^e^	2.31
PPC_sol_	N/A^e^	14.6 ± 1.8	N/A^e^	1.75 ± 0.15	1.00	N/A^e^	N/A^e^	2.22
PVF_sol_	175,000^a^	14.0 ± 1.8	5.814 × 10^–9^	0.95 ± 0.11	0.95	5.91 × 10^–11^	0.603	1.64
WPI_sol_	36,600^b^	15.2 ± 0.5	2.428 × 10^–9^	1.00	1.00	9.82 × 10^–11^	1.000	1.79

a From SEC analysis.

b Calculations based on the theoretical *M*_w_ of β-lactoglobulin dimer, P02754, UniProt database.^25^

c Experimental ζ-potential.

d From ANSA.

e Not applicable.

### 3.2 Effect of Legumin-to-Vicilin Ratio on the Interfacial Properties of Pea

**Figure 3:**
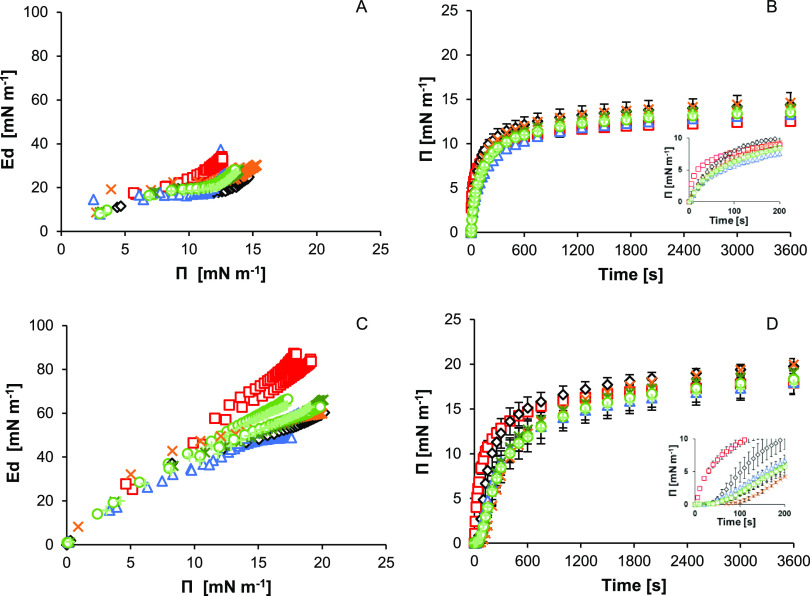
Elastic modulus (*E*_d_) as a function of surface pressure (A, C), results of duplicate measurements shown as individual markers. Surface pressure as function of time (B, D), marker indicates mean of duplicates ± standard deviation. Samples: PLF_sol_ (orange crosses), PVF_sol_ (blue triangle), PPC_sol_ (black diamonds), PPC_sim_ (light green plus), LV_5050_ (medium green circles), LV_7030_ (dark green asterisks) and WPI_sol_ (red boxes). A and B measured at the oil–water and C and D at the air–water interface. The inset shows the surface pressure at 0–200 s.

At both the oil–water and air–water interface there were no relevant significant differences between the interfacial properties of the pea protein samples (Figure 3). In addition, WPI_sol_ had a larger increase in surface pressure (dΠ/d*t*) and a higher surface dilatational elastic modulus (*E*_d_) than the pea protein samples at both the oil–water and air–water interface. For these samples, the interfacial properties at the air–water interface were a good indication of the protein interfacial properties at the oil–water interface. As a consequence, it was assumed that the oil–water interfacial properties could be compared to the air–water interfacial properties reported in the literature (and vice versa). The curves of *E*_d_ versus surface pressures (Π) for adsorption at the oil–water interface were similar for all pea protein samples (Figure 3A). The *E*_d_/Π curves of the pea proteins samples differed from that of WPI_sol._ The similar *E*_d_/Π curves for the pea protein samples suggested that their equation of states (Π/Γ) were also similar. Therefore, a similar dΠ/d*t* was interpreted as a similar adsorption rate (dΓ/d*t*). The *E*_d_/Π for the adsorption of pea protein samples at the oil–water interface was not linear. In contrast, at the air–water interface pure protein systems are known to show linear Ed/Π relationships until 10–15 mN m^–1^.^34,46^ The maximum *E*_d_ reached for the adsorption of the pea protein samples at the oil–water interface was lower: *E*_d_ 25–30 mN m^–1^/Π 13–15 mN m^–1^_,_ than that of WPI_sol_: *E*_d_ 33 mN m^–1^/Π 13 mN m^–1^_._ The lower final elastic moduli of the pea protein samples than of WPI_sol_ could be an indication of the presence of low molecular weight surfactants. The presence of low molecular weight surfactants is known to decrease the *E*_d_.^47^ The initial increase of surface pressure at the oil–water interface was similar for all pea protein samples (15.8 ± 2.4 s to reach 2 mN m^–1^, Figure 3B). For WPI_sol_, the initial increase in surface pressure at the oil–water interface was approximately 6 times faster (2.7 ± 0.9 s to reach 2 mN m^–1^) than that of the pea protein samples. The final surface pressure of the pea proteins samples, approximately 14 mN m^–1^, was similar to that of WPI_sol_. For WPI and commercial PPC, similar surface pressures were reported after 3600 s: 15 and 17 mN m^–1^, respectively; at 0.1 g L^–1^, 10 mM sodium phosphate buffer, pH 7.0.^48^ The dΠ/d*t* was faster for the samples at the oil–water interface than for the corresponding samples at the air–water interface. The difference in dΠ/d*t* between the oil–water and air–water interface has been reported by other authors.^49^

As described above, the ζ-potentials and *k*_adsorb_ of the pea protein samples were similar to WPI_sol_. The *k*_adsorb_ for WPI_sol_ and the pea protein samples was approximately 1, assuming that there was no barrier of adsorption for the proteins to the interface. Therefore, at *t* = 0 s, all proteins from the sub-surface layer were adsorbed to the interface, and the protein concentration (*C*_p_) in the sub-surface layer was equal to 0 g L^–1^. This resulted in a concentration difference between the sub-surface layer and the bulk, which in turn induced a diffusion process. Since the radii of the pea legumin and vicilin were 2.2 times larger than that of the β-lactoglobulin in WPI_sol_, their diffusion coefficient was 2.2 times smaller. So, the diffusion of β-lactoglobulin in WPI_sol_ to the interface was faster than for the proteins in the pea protein samples. This explains, at least in part, the difference in dΠ/d*t* of the pea protein samples and the WPI_sol_. Based on the higher dΠ/d*t*, WPI_sol_ was expected to have a higher efficiency to stabilize emulsion droplets than the pea protein samples. Therefore, WPI_sol_ was expected to have a lower critical protein concentration (*C*_cr_). Further, no effect of the L:V ratio on the interfacial properties was observed. Therefore, based on these findings no effect of the L:V ratio on the *C*_cr_ was expected.

### 3.3 Effect of Legumin-to-Vicilin Ratio in the Emulsifying Properties of Pea Proteins

**Figure 4:**
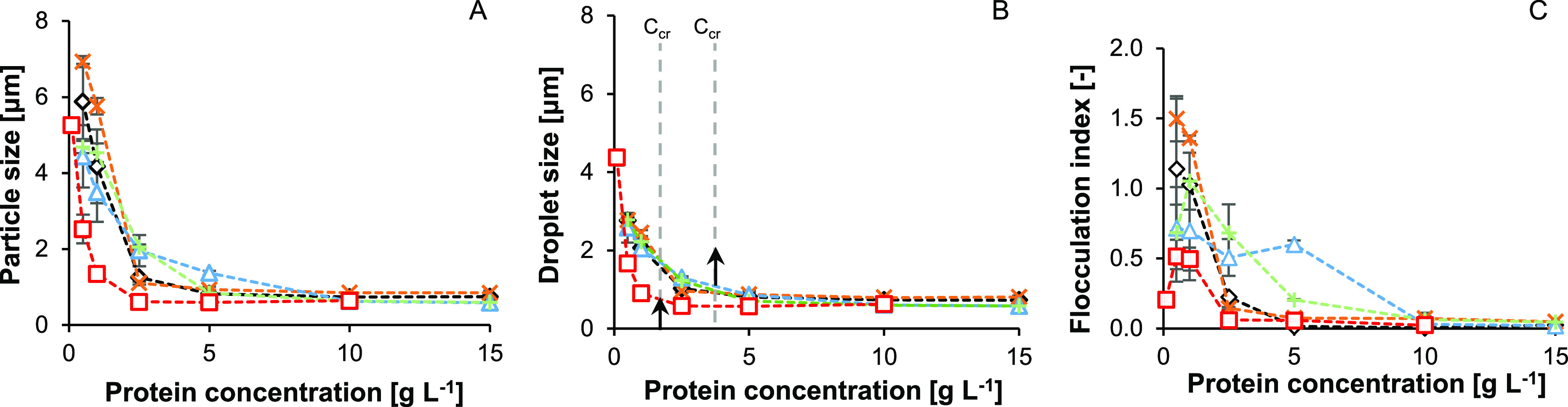
Volume–surface average diameter (*d*_3,2_) as function of protein concentration of emulsions stabilized with PLF_sol_ (orange crosses), PVF_sol_ (blue triangles), PPC_sol_ (black diamonds), PPC_sim_ (green plus), and WPI_sol_ (red boxes) ± standard deviation. Measured without (A) and with (B) SDS and flocculation index (C). Dashed lines are to guide the eye.

**Figure 5:**
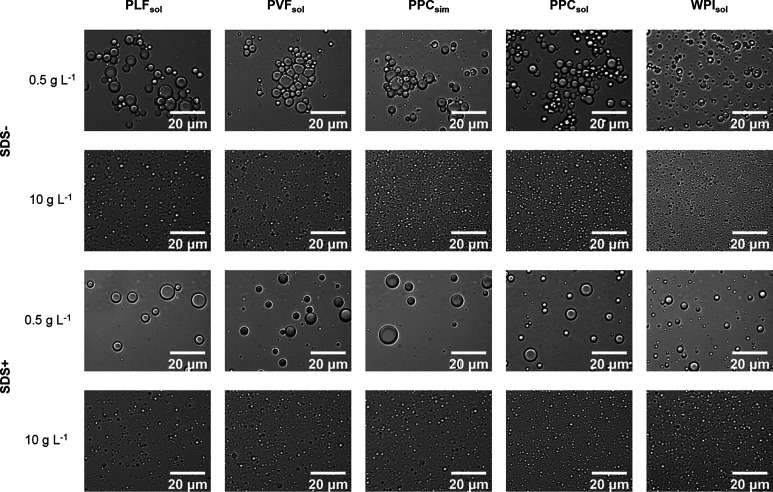
Light microscopy pictures of emulsions stabilized with PLF_sol_, PVF_sol_, PPC_sim_, PPC_sol_, and WPI_sol_ at 0.5 and 10 g L^–1^, with and without the addition of SDS.

The effect of L:V ratio on the emulsifying properties of pea proteins samples was determined by analyzing the individual emulsion droplet and flocculate size as function of *C*_p_. The curves of the emulsion particle size against *C*_p_ were similar for all pea protein samples (Figure 4A). The emulsion particle sizes of the WPI_sol_ emulsions were smaller than those of the pea protein emulsions at the same *C*_p_ for concentrations below 10 g L^–1^. The emulsion particle sizes decreased with increasing *C*_p_ for all samples. PLF_sol_ and PPC_sol_ emulsions flocculated at protein concentrations below 5.0 g L^–1^, and PPC_sim_ and PVF_sol_ emulsions at protein concentrations below 10.0 g L^–1^ (Figure 4C). WPI_sol_ emulsions flocculated at protein concentrations below 2.5 g L^–1^ (Figure 4C). Light microscopy images confirmed the presence of flocculated emulsion droplets at 0.5 g L^–1^ protein (Figure 5). Flocculation of commercial^16^ and non-commercial pea proteins^18^ was reported at similar conditions (pH 7.0, low ionic strength). The extent and the concentration at which flocculation occurred were lower for the WPI_sol_ emulsions than for the pea protein emulsions. No trend was observed between the L:V ratio and the flocculation behavior, or the presence of non-protein compounds, 9.5–25% (w/w, dry matter), and flocculation behavior. Overall, the FI decreased with increasing *C*_p_. The effect of *C*_p_ on emulsion flocculation was observed and described by Delahaije et al.^15^ The authors showed that emulsions prepared with 5 g L^–1^ β-lactoglobulin had smaller droplet sizes than those prepared with 1.5, 2.0, and 2.5 g L^–1^.The addition of extra protein to a β-lactoglobulin, ovalbumin, or patatin-rich emulsion in the protein-poor regime resulted in increased stability toward flocculation at high ionic strength. Delahaije et al.^15^ explained this effect by the higher maximum adsorbed amount of protein (Γ_max_) at high ionic strength. As a result of the higher Γ_max_ more protein was needed to cover the interface. Therefore, the presence of excess protein in the bulk at *C*_p_ > critical protein concentration (*C*_cr_), increased the stability toward flocculation. Another proposed explanation for the stabilizing effect of excess protein was the addition of a steric or electrostatic repulsive force, by the formation of multilayers of due to non-adsorbed proteins in the bulk.

The curves of the individual emulsion droplet sizes versus the *C*_p_ were similar for all pea protein emulsions (Figure 4B). A minimal droplet size of approximately 0.67 μm was reached at a *C*_cr_ between 2.5 and 5.0 g L^–1^. The L:V ratio did not affect the emulsion particle or individual droplet size, *d*_3,2_. Therefore, reported differences in the emulsion properties of pea proteins under similar conditions^1−3,17^ were not caused by differences in the L:V ratio. No trend was observed between the presence of non-protein compounds, 9.5–25% (w/w, dry matter), and emulsion droplet size. The *C*_cr_ of WPI_sol_ was lower (*C*_cr_ = 1.0–2.5 g L^–1^) than the *C*_cr_ of the pea protein samples. Light microscopy images confirmed that the emulsion droplets stabilized by WPI_sol_ were smaller than those stabilized by the pea protein samples at 0.5 g L^–1^ protein (Figure 5). This was in line with the interfacial properties of the proteins, as WPI_sol_ had a higher dΠ/d*t* than the pea protein samples. As explained above, the differences in dΠ/d*t* were mainly caused by differences in the radii of the proteins.

### 3.4 Predicting the Effect of Legumin-to-Vicilin Ratio on the *d*_3,2_ vs *C*_p_

**Figure 6:**
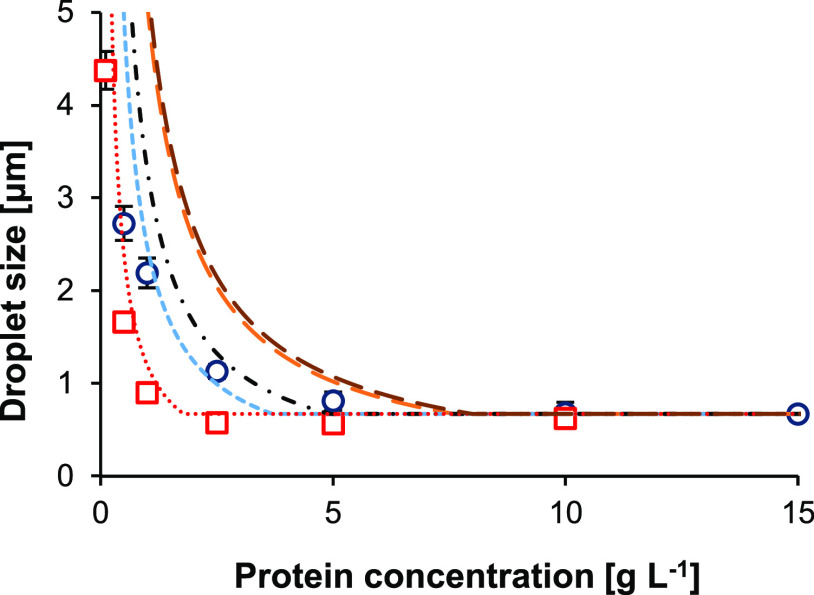
Volume–surface average diameter (*d*_3,2_) as a function of protein concentration of emulsions stabilized with pea protein samples, represented as average of all pea protein samples per *C*_p_ (black circles), or WPI_sol_ (red boxes). All samples were measured with SDS, error bars represent standard deviation. Theoretical prediction of PLF_sol_ (long dashed orange line) and PLF_sol,aggr_ (long dashed dark red line) assuming 24% of legumin is present as aggregates (600 kDa) and 76% of legumin is present as hexamers, PPC_sol_ (dash-dot black line), PVF_sol_ (dashed blue line), and WPI_sol_ (dotted red line).

The molecular properties of the protein (*R*_eff_, ζ-potential, relative hydrophobicity) were used to theoretically predict the volume–surface average diameter (*d*_3,2,theo_) (Table 2). As described above, the main difference amongst the proteins was the radii of the protein. Therefore, the relative diffusion coefficient (*D*′) was added as a parameter in the extended surface coverage model, described in this study. The importance of a diffusion coefficient in describing protein interfacial properties has been shown by others.^50−52^ However, until now the diffusion of proteins was not explicitly taken into account in the model to predict *d*_3,2_ for single protein systems.^9^ The *d*_3,2,theo_ was calculated from eq 2, using the oil fraction (Φ_oil_), the maximum adsorbed amount of protein on the interface (Γ_max,theo_), the protein concentration (*C*_p_), the adsorption rate constant (*k*_adsorb_), and the relative diffusion coefficient (*D*′). For the calculation of the maximum adsorbed amount of protein on the interface a saturation coverage (θ_∞_) of 0.547 was used. As a θ_∞_ of 0.547 is sufficient to prevent the coalescence of emulsion droplets prepared from single protein systems, it was also considered to be sufficient for mixed protein systems even though the experimental value can be higher. The extended model, described in this paper, predicted a theoretical *C*_cr_ (*C*_cr,theo_) of 7.6, 5.2, 5.0, 3.7, and 1.8 g L^–1^ for PLF_sol_, PPC_sol_, PVF_sol_, and WPI_sol_, respectively (Figure 6). For PPC_sol_, PVF_sol_, and WPI_sol_, these values were close to or within the range of the experimentally determined *C*_cr_. For PLF_sol_, the *C*_cr,theo_ was slightly higher than the experimental *C*_cr_. A possible explanation for the overestimation is that the configuration of the hexameric legumin at the interface was estimated incorrectly, resulting in a too high value for Γ_max,theo_. The *C*_cr,theo_ of PLF_sol_, assuming 100% hexameric legumin, and PLF_sol,aggr_, assuming 24% aggregated legumin and 76% hexameric legumin, were similar to each other: 7.6 and 8.0 g L^–1^ for PLF_sol_ and PLF_sol,aggr_, respectively. This confirmed that the presence of 24% aggregated legumin did not have a large effect on the theoretical prediction. Overall, the curves of the *d*_3,2,theo_ vs *C*_p_ were similar to the respective curves of *d*_3,2_ vs *C*_p_.

Concluding, despite the molecular differences between the pea legumin (PLF_sol_) and pea vicilin fraction (PVF_sol_), the interfacial properties at the oil–water interface (*E*_d_–Π and Π–*t* curves) were similar for these samples. PLF_sol_ and PVF_sol_ have similar emulsifying properties (emulsion droplet or particle size versus *C*_p_) at pH 7.0 (conductivity buffer 2.3 mS cm^–1^). This disproves the generally held idea that vicilin has better emulsifying properties due to its more flexible structure. In addition, it shows that the variations in the emulsifying properties of different pea protein samples reported in the literature cannot be explained by differences in the L:V ratio. Both PLF_sol_ and PVF_sol_ were less efficient than whey protein isolate (WPI_sol_) in stabilizing the newly formed emulsion droplets. This difference was attributed to the differences in diffusion coefficients. The relative diffusion coefficient was included in the extended surface coverage model described in this paper. With this addition, the *d*_3,2_ versus *C*_p_ of the pure and mixed pea protein samples was well described by the model, based on the molecular properties of the proteins.

## Supporting Information Available

Surface pressure as a function of time at 0−200 s of PLF_sol_, PVF_sol_, PPC_sol_, PPC_sim_, LV_5050_, LV_7030_, and WPI_sol_ (PDF)

## Abbreviations and Symbols

L:V: legumin-to-vicilin
*d*_3,2_: volume-surface average diameter
*C*_p_: protein concentration
PLF_sol_: soluble pea legumin fraction
PVF_sol_: soluble pea vicilin fraction
Γ_max,theo_: theoretical maximum adsorbed amount of protein on the interface
WPI_sol_: soluble whey protein isolate
EAI: emulsifying activity index
PPI: pea protein isolate
EC: emulsifying capacity
Φ_oil_: oil fraction
*C*_cr_: experimental critical protein concentration
FI: flocculation index
PPC: pea protein concentrate
PLF: pea legumin fraction
PVF: pea vicilin fraction
WPI: whey protein isolate
SDS-PAGE: sodium dodecyl sulphate polyacrylamide gel electrophoresis
MQ: Milli-Q water
PPC^–20^: pea protein concentrate in water adjusted to pH 8.0 and stored at −20 °C prior to freeze drying or further fractionation
RT: room temperature
LV_7030_: PLF_sol_ and PVF_sol_ mixed in a 70:30 ratio (v/v)
LV_5050_: PLF_sol_ and PVF_sol_ mixed in a 50:50 ratio (v/v)
PPC_sim_: PLF_sol_ and PVF_sol_ mixed in a 30:70 ratio (v/v)
ANSA: 8-anilino-1-naphthalenesulfonic acid
γ: surface tension
Π: surface pressure
*E*_d_: elastic modulus
*d*_3,2,theo_: theoretical volume-surface average diameter
*D*′: relative diffusion coefficient
*k*_adsorb_: adsorption rate constant
*Q*_h_: relative exposed hydrophobicity
*k*_b_: Boltzmann constant
*T*: temperature
η: viscosity
*R*_p_: radius of the protein
θ_∞_: saturation coverage
*N*_a_: Avogadro’s constant
*M*_w_: molecular weight
*R*_eff_: effective radius of the protein
Γ_max_: maximum adsorbed amount of protein on the interface
*C*_cr,theo_: theoretical critical protein concentration

## References

[ref1] Barać M. B.; Pešić M. B.; Stanojević S. P.; Kostić Ž. A.; Bivolarević V. Comparative study of the functional properties of three legume seed isolates: adzuki, pea and soy bean. J. Food Sci. Technol. 2015, 52, 2779–2787. 10.1007/s13197-014-1298-6. 25892775 PMC4397294

[ref2] Barać M. B.; Čabrilo S.; Pešić M. B.; Stanojević S. P.; Pavlićević M.; Maćej O.; Ristić N. Functional properties of pea (*Pisum sativum*, L.) protein isolates modified with chymosin. Int. J. Mol. Sci. 2011, 12, 8372–8387. 10.3390/ijms12128372. 22272078 PMC3257075

[ref3] Barać M. B.; Čabrilo S.; Pešić M. B.; Stanojević S. P.; Žilić S.; Maćej O.; Ristić N. Profile and functional properties of seed proteins from six pea (*Pisum sativum*) genotypes. Int. J. Mol. Sci. 2010, 11, 4973–4990. 10.3390/ijms11124973. 21614186 PMC3100834

[ref4] Casey R.; Sharman J. E.; Wright D. J.; Bacon J. R.; Guldager P. Quantitative variability in *Pisum* seed globulins: its assessment and significance. Plant Foods Hum. Nutr. 1982, 31, 333–346. 10.1007/BF01094045.

[ref5] Gueguen J.; Barbot J. Quantitative and qualitative variability of pea (*Pisum sativum* L.) protein composition. J. Sci. Food Agric. 1988, 42, 209–224. 10.1002/jsfa.2740420304.

[ref6] Schroeder H. E. Quantitative studies on the cotyledonary proteins in the genus *Pisum*. J. Sci. Food Agric. 1982, 33, 623–633. 10.1002/jsfa.2740330707. 7120923

[ref7] Tzitzikas E. N.; Vincken J.-P.; de Groot J.; Gruppen H.; Visser R. G. F. Genetic variation in pea seed globulin composition. J. Agric. Food Chem. 2006, 54, 425–433. 10.1021/jf0519008. 16417300

[ref8] Vreeke G. J.; Meijers M. G.; Vincken J.-P.; Wierenga P. A. Towards absolute quantification of protein genetic variants in Pisum sativum extracts. Anal. Biochem. 2023, 665, 115048 10.1016/j.ab.2023.115048. 36657509

[ref9] Delahaije R. J. B. M.; Gruppen H.; Giuseppin M. L. F.; Wierenga P. A. Towards predicting the stability of protein-stabilized emulsions. Adv. Colloid Interface Sci. 2015, 219, 1–9. 10.1016/j.cis.2015.01.008. 25704489

[ref10] Swift C. E.; Fryar A. J.; Lockett C. Comminuted meat emulsions - Capacity of meats for emulsifying fat. Food Technol. 1961, 15, 468.

[ref11] Halling P. J.; Walstra P. Protein-stabilized foams and emulsions. Crit. Rev. Food Sci. Nutr. 1981, 15, 155–203. 10.1080/10408398109527315. 7023848

[ref12] Pearce K. N.; Kinsella J. E. Emulsifying properties of proteins: evaluation of a turbidimetric technique. J. Agric. Food Chem. 1978, 26, 716–723. 10.1021/jf60217a041.

[ref13] Cameron D. R.; Weber M. E.; Idziak E. S.; Neufeld R. J.; Cooper D. G. Determination of interfacial areas in emulsions using turbidimetric and droplet size data: correction of the formula for emulsifying activity index. J. Agric. Food Chem. 1991, 39, 655–659. 10.1021/jf00004a005.

[ref14] Tcholakova S.; Denkov N. D.; Sidzhakova D.; Ivanov I. B.; Campbell B. Interrelation between drop size and protein adsorption at various emulsification conditions. Langmuir 2003, 19, 5640–5649. 10.1021/la034411f.

[ref15] Delahaije R. J. B. M.; Wierenga P. A.; Van Nieuwenhuijzen N. H.; Giuseppin M. L. F.; Gruppen H. Protein concentration and protein-exposed hydrophobicity as dominant parameters determining the flocculation of protein-stabilized oil-in-water emulsions. Langmuir 2013, 29, 11567–11574. 10.1021/la401314a. 23859264

[ref16] Chen M.; Lu J.; Liu F.; Nsor-Atindana J.; Xu F.; Goff H. D.; Ma J.; Zhong F. Study on the emulsifying stability and interfacial adsorption of pea proteins. Food Hydrocolloids 2019, 88, 247–255. 10.1016/j.foodhyd.2018.09.003.

[ref17] Kimura A.; Fukuda T.; Zhang M.; Motoyama S.; Maruyama N.; Utsumi S. Comparison of Physicochemical Properties of 7S and 11S Globulins from Pea, Fava Bean, Cowpea, and French Bean with Those of Soybean French Bean - 7S Globulin Exhibits Excellent Properties. J. Agric. Food Chem. 2008, 56, 10273–10279. 10.1021/jf801721b. 18828597

[ref18] Liang H.; Tang C. pH-dependent emulsifying properties of pea [*Pisum sativum* (L.)] proteins. Food Hydrocolloids 2013, 33, 309–319. 10.1016/j.foodhyd.2013.04.005.

[ref19] Dagorn-Scaviner C.; Gueguen J.; Lefebvre J. A comparison of interfacial behaviours of pea (Pisum sativum L.) legumin and vicilin at air/water interface. Food/Nahrung 1986, 30, 337–347. 10.1002/food.19860300332.

[ref20] Lam A.; Can Karaca A.; Tyler R.; Nickerson M. Pea protein isolates: Structure, extraction, and functionality. Food Rev. Int. 2018, 34, 126–147. 10.1080/87559129.2016.1242135.

[ref21] O’Kane F. E.; Happe R. P.; Vereijken J. M.; Gruppen H.; Van Boekel M. A. Characterization of pea vicilin. 1. Denoting convicilin as the α-subunit of the *Pisum* vicilin family. J. Agric. Food Chem. 2004, 52, 3141–3148. 10.1021/jf035104i. 15137866

[ref22] Berton C. C.; Genot C.; Ropers M. H. Quantification of unadsorbed protein and surfactant emulsifiers in oil-in-water emulsions. J. Colloid Interface Sci. 2011, 354, 739–748. 10.1016/j.jcis.2010.11.055. 21167495

[ref23] Maldonado-Valderrama J.; Woodward N. C.; Gunning A. P.; Ridout M. J.; Husband F. A.; Mackie A. R.; Morris V. J.; Wilde P. J. Interfacial characterization of β-lactoglobulin networks: Displacement by bile salts. Langmuir 2008, 24, 6759–6767. 10.1021/la800551u. 18533634

[ref24] Butré C. I.; Wierenga P. A.; Gruppen H. Effects of ionic strength on the enzymatic hydrolysis of diluted and concentrated whey protein isolate. J. Agric. Food Chem. 2012, 60, 5644–5651. 10.1021/jf301409n. 22583537

[ref25] UniProtKB. http://www.uniprot.org/ (accessed Jun 4, 2021).

[ref26] Englyst H. N.; Cummings J. H. Simplified method for the measurement of total non-starch polysaccharides by gas-liquid chromatography of constituent sugars as alditol acetates. Analyst 1984, 109, 937–942. 10.1039/an9840900937. 6283946

[ref27] Blumenkrantz N.; Asboe-Hansen G. New method for quantitative determination of uronic acids. Anal. Biochem. 1973, 54, 484–489. 10.1016/0003-2697(73)90377-1. 4269305

[ref28] Kiskini A.; Vissers A.; Vincken J.-P.; Gruppen H.; Wierenga P. A. Effect of plant age on the quantity and quality of proteins extracted from sugar beet (Beta vulgaris L.) leaves. J. Agric. Food Chem. 2016, 64, 8305–8314. 10.1021/acs.jafc.6b03095. 27750423

[ref29] Jurak E.; Punt A. M.; Arts W.; Kabel M. A.; Gruppen H. Fate of carbohydrates and lignin during composting and mycelium growth of Agaricus bisporus on wheat straw based compost. PLoS One 2015, 10, e0138909 10.1371/journal.pone.0138909. 26436656 PMC4593547

[ref30] Szymanowska U.; Jakubczyk A.; Baraniak B.; Kur A. Characterisation of lipoxygenase from pea seeds (*Pisum sativum* var. Telephone L.). Food Chem. 2009, 116, 906–910. 10.1016/j.foodchem.2009.03.045.

[ref31] O’Kane F. E. Molecular characterisation and heat-induced gelation of pea vicilin and legumin. Ph.D. Thesis, Wageningen University, 2004.

[ref32] Jachimska B.; Wasilewska M.; Adamczyk Z. Characterization of globular protein solutions by dynamic light scattering, electrophoretic mobility, and viscosity measurements. Langmuir 2008, 24, 6866–6872. 10.1021/la800548p. 18512882

[ref33] Haskard C. A.; Li-Chan E. C. Y. Hydrophobicity of bovine serum albumin and ovalbumin determined using uncharged (PRODAN) and anionic (ANS-) fluorescent probes. J. Agric. Food Chem. 1998, 46, 2671–2677. 10.1021/jf970876y.

[ref34] Delahaije R. J. B. M.; Gruppen H.; Giuseppin M. L. F.; Wierenga P. A. Quantitative description of the parameters affecting the adsorption behaviour of globular proteins. Colloids Surf., B 2014, 123, 199–206. 10.1016/j.colsurfb.2014.09.015. 25280607

[ref35] Sridharan S.; Meinders M. B.; Bitter J. H.; Nikiforidis C. V. On the emulsifying properties of self-assembled pea protein particles. Langmuir 2020, 36, 12221–12229. 10.1021/acs.langmuir.0c01955. 32988196 PMC7586397

[ref36] Talbot J.; Tarjus G.; Van Tassel P. R.; Viot P. From car parking to protein adsorption: an overview of sequential adsorption processes. Colloids Surf., A 2000, 165, 287–324. 10.1016/S0927-7757(99)00409-4.

[ref37] Tandang-Silvas M. R. G.; Fukuda T.; Fukuda C.; Prak K.; Cabanos C.; Kimura A.; Itoh T.; Mikami B.; Utsumi S.; Maruyama N. Conservation and divergence on plant seed 11S globulins based on crystal structures. Biochim. Biophys. Acta, Proteins Proteomics 2010, 1804, 1432–1442. 10.1016/j.bbapap.2010.02.016. 20215054

[ref38] Sridharan S.; Meinders M. B.; Bitter J. H.; Nikiforidis C. V. Pea flour as stabilizer of oil-in-water emulsions: Protein purification unnecessary. Food Hydrocolloids 2020, 101, 105533 10.1016/j.foodhyd.2019.105533.

[ref39] Nikolopoulou D.; Grigorakis K.; Stasini M.; Alexis M.; Iliadis K. Differences in chemical composition of field pea (*Pisum sativum*) cultivars: Effects of cultivation area and year. Food Chem. 2007, 103, 847–852. 10.1016/j.foodchem.2006.09.035.

[ref40] Redondo-Cuenca A.; Villanueva-Suárez M.; Rodríguez-Sevilla M.; Mateos-Aparicio I. Chemical composition and dietary fibre of yellow and green commercial soybeans (Glycine max). Food Chem. 2007, 101, 1216–1222. 10.1016/j.foodchem.2006.03.025.

[ref41] De Almeida Costa G. E.; Da Silva Queiroz-Monici K.; Reis S. M. P. M.; De Oliveira A. C. Chemical composition, dietary fibre and resistant starch contents of raw and cooked pea, common bean, chickpea and lentil legumes. Food Chem. 2006, 94, 327–330. 10.1016/j.foodchem.2004.11.020.

[ref42] Cui L.; Bandillo N.; Wang Y.; Ohm J.-B.; Chen B.; Rao J. Functionality and structure of yellow pea protein isolate as affected by cultivars and extraction pH. Food Hydrocolloids 2020, 108, 106008 10.1016/j.foodhyd.2020.106008.

[ref43] Da Silva A. M. M.; Almeida F. S.; Sato A. C. K. Functional characterization of commercial plant proteins and their application on stabilization of emulsions. J. Food Eng. 2021, 292, 110277 10.1016/j.jfoodeng.2020.110277.

[ref44] Gueguen J.; Chevalier M.; Barbot J.; Schaeffer F. Dissociation and aggregation of pea legumin induced by pH and ionic strength. J. Sci. Food Agric. 1988, 44, 167–182. 10.1002/jsfa.2740440208.

[ref45] Gatehouse J. A.; Croy R. R. D.; Morton H.; Tyler M.; Boulter D. Characterisation and subunit structures of the vicilin storage proteins of pea (*Pisum sativum* L.). Eur. J. Biochem. 1981, 118, 627–633. 10.1111/j.1432-1033.1981.tb05565.x. 7297569

[ref46] Teuling E.; Schrama J. W.; Gruppen H.; Wierenga P. A. Characterizing emulsion properties of microalgal and cyanobacterial protein isolates. Algal Res. 2019, 39, 101471 10.1016/j.algal.2019.101471.

[ref47] Lech F. J.; Meinders M. B. J.; Wierenga P. A.; Gruppen H. Comparing foam and interfacial properties of similarly charged protein–surfactant mixtures. Colloids Surf., A 2015, 473, 18–23. 10.1016/j.colsurfa.2014.12.063.

[ref48] Hinderink E. B. A.; Sagis L.; Schroën K.; Berton-Carabin C. C. Behavior of plant-dairy protein blends at air-water and oil-water interfaces. Colloids Surf., B 2020, 192, 111015 10.1016/j.colsurfb.2020.111015. 32416469

[ref49] Beverung C. J.; Radke C. J.; Blanch H. W. Protein adsorption at the oil/water interface: characterization of adsorption kinetics by dynamic interfacial tension measurements. Biophys. Chem. 1999, 81, 59–80. 10.1016/S0301-4622(99)00082-4. 10520251

[ref50] Ward A. F. H.; Tordai L. Time-dependence of boundary tensions of solutions I. The role of diffusion in time-effects. J. Chem. Phys. 1946, 14, 453–461. 10.1063/1.1724167.

[ref51] Ravera F.; Liggieri L.; Steinchen A. Sorption kinetics considered as a renormalized diffusion process. J. Colloid Interface Sci. 1993, 156, 109–116. 10.1006/jcis.1993.1088.

[ref52] Wierenga P. A.; Meinders M. B. J.; Egmond M. R.; Voragen A. G. J.; de Jongh H. H. J. Quantitative description of the relation between protein net charge and protein adsorption to air– water interfaces. J. Phys. Chem. B 2005, 109, 16946–16952. 10.1021/jp050990g. 16853156

