# Supplementary material for: Effect of Pea Legumin-to-Vicilin Ratio on the Protein Emulsifying Properties: Explanation in Terms of Protein Molecular and Interfacial Properties
Source: J Agric Food Chem. 2023 Jul 11;71(29):11228–38. doi: 10.1021/acs.jafc.3c01589 (PMC10375591; doi:10.1021/acs.jafc.3c01589)
Supplement: Supplementary file 1 — jf3c01589_si_001.pdf [file jf3c01589_si_001.pdf]

Supplementary information to

The effect of pea legumin-to-vicilin ratio on the protein emulsifying properties: explanation in terms of protein molecular and interfacial properties.

Maud G.J. Meijers[a, b], Marcel B.J. Meinders[a,c], Jean-Paul Vincken[b], Peter A. Wierenga[b]

[a] TiFN, Nieuwe Kanaal 9A, 6709 PA, Wageningen, The Netherlands

[b] Laboratory of Food Chemistry, Wageningen University and Research, Bornse Weilanden 9, 6708 WG, Wageningen, The Netherlands.

[c] Food and Biobased Research, Wageningen University and Research, Bornse Weilanden 9, 6708 WG, Wageningen, The Netherlands.

Corresponding author:

Peter Wierenga, peter.wierenga@wur.nl

Content

Figure 1. Surface pressure as function of time at 0-200 s of PLF_{sol} (×), PVF_{sol} (△), PPC_{sol} (◇), PPC_{sim} (+), LV_{5050} (○), LV_{7030} (✱) and WPI_{sol} (□). The marker indicates mean of duplicates ± standard deviation. A is measured at the oil-water and B at the air-water interface.

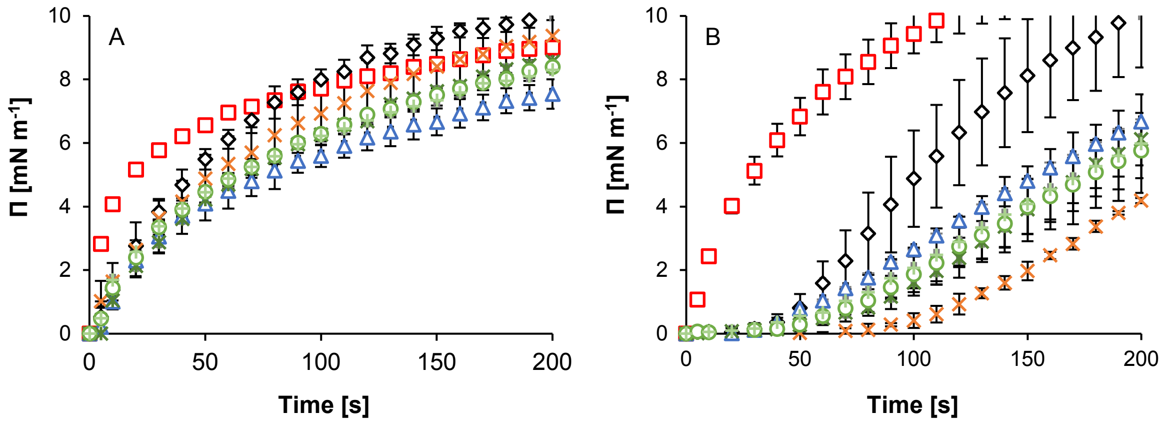

Figure 1. Surface pressure as function of time at 0-200 s of PLF$_{sol}$ (×), PVF$_{sol}$ (△), PPC$_{sol}$ (◇), PPC$_{sim}$ (+), LV$_{5050}$ (○), LV$_{7030}$ (✱) and WPI$_{sol}$ (□). The marker indicates mean of duplicates ± standard deviation. A is measured at the oil-water and B at the air-water interface.
